# Fibronectin precoating wound bed enhances the therapeutic effects of autologous epidermal basal cell suspension for full-thickness wounds by improving epidermal stem cells’ utilization

**DOI:** 10.1186/s13287-019-1236-7

**Published:** 2019-09-11

**Authors:** Peng Wang, Zhicheng Hu, Xiaoling Cao, Shaobin Huang, Yunxian Dong, Pu Cheng, Hailin Xu, Bin Shu, Julin Xie, Jun Wu, Bing Tang, Jiayuan Zhu

**Affiliations:** Department of Burn Surgery, The First Affiliated Hospital of Sun Yat-sen University, No.58, Zhongshan 2nd Road, Guangzhou, 510080 China

**Keywords:** FN, Collagen IV, Autologous EBC suspension therapy, ESCs, Full-thickness wounds

## Abstract

**Background:**

Autologous epidermal basal cell suspension therapy has been proven to be one of the most effective treatments for full-thickness wounds. However, we found there remain obvious defects that significantly confined the utilization and function of the epidermal basal cells (EBCs), especially the epidermal stem cells (ESCs) in it. This study investigated whether precoating fibronectin (FN) on the wound bed before spraying EBCs could overcome these defects and further explored its possible mechanisms.

**Methods:**

In the in vitro study, EBCs were isolated from the donor skin of patients who needed skin grafting. Different concentrations of FN were used to precoat culture dishes before cell culture; the adherent efficiency, proliferation and migration ability of ESCs were analyzed and compared with traditional collagen IV precoating.

In the in vivo study, Sprague–Dawley (SD) rats with full-thickness skin wounds were selected as full-thickness wounds’ model. For the experiment groups, 20 μg/ml FN was precoated on the wound bed 10 min before EBC spray. The quality of wound healing was estimated by the residual wound area rate, wound healing time, and hematoxylin and eosin (H&E) staining. Expression of ESC markers, neovascular markers, inflammation markers, and collagen formation and degradation markers was elucidated by immunohistochemistry (IHC), immunofluorescence (IF), western blot (WB), and RT-qPCR analysis.

**Results:**

The in vitro study showed that the dishes precoated with 20 μg/ml FN had a similar adherent efficiency and colony formation rate with collagen IV, but it could improve the proliferation and migration of ESCs significantly. Similarly, in the in vivo study, precoating FN on wound bed before EBC spray also significantly promote wound healing by improving ESCs’ utilization efficiency, promoting angiogenesis, decreasing inflammations, and regulating collagen formation and degradation.

**Conclusion:**

FN precoating wound bed before EBC spray could significantly promote full-thickness wound healing by improving the utilization and function of the ESCs and further by promoting angiogenesis, decreasing inflammations, and regulating collagen formation and degradation.

**Graphical abstract:**

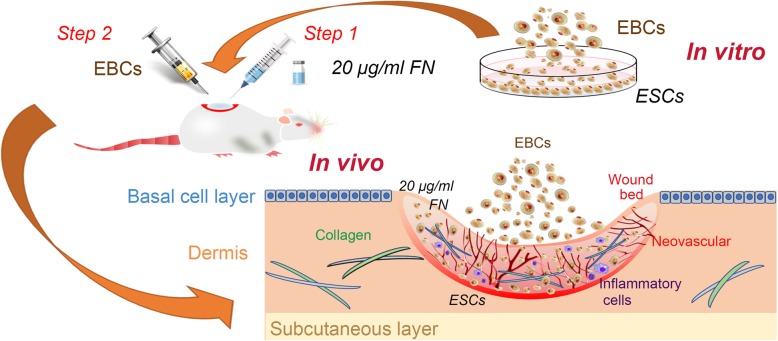

## Background

Full-thickness wounds are a common and serious problem worldwide that affects patients of various wound types caused by burns, traumatic injuries, and diabetes [1]. Although various therapeutic interventions have been attempted to treat full-thickness wounds, current wound care remains far from ideal healing and brings a heavy burden to society [2]. Thus, effective skin wound healing becomes a major concern for global healthcare and requires further exploration.

As we all know, the optimal healing of a full-thickness wound requires a well-orchestrated integration of the complex biological and molecular events involving multiple cell types, extracellular matrix (ECM), and growth factors [3]. Despite the wide range of clinically available skin substitutes and the different therapeutic alternatives, delayed healing and scarring are frequently observed. In recent years, the approach of autologous EBC transplantation has emerged as an alternative to improve the outcome of healing and achieved exciting progress [4]. Our previous randomized clinical trial displayed that using the combination of split-thickness autologous skin grafts and autologous EBCs to treat chronic wound could significantly promote wound healing, improve long-term esthetic appearance, and reduce clinical complications than split-thickness skin grafts alone [5]. However, as the research progressed, we found there were three major defects in this treatment remained to conquer. Firstly, the sprayed EBCs could not implantation on the wound bed tightly, and many cells were lost and wasted by flowing away. Secondly, the EBCs planted on the wound surface were disorganized and could not play their role effectively. Thirdly, the residual bacteria in the wound bed might easily contaminate cells and lead them to die. Since these three defects significantly confined the utilization and function of the autologous EBCs, we hypothesized that overcoming these critical problems could further improve the therapeutic effect of full-thickness wounds.

It has been proven that the main obstacle for the full-thickness wound healing is the absence of extracellular matrix and keratinocytes, especially ESCs [6]. In our previous study, we have confirmed that there are 60% keratinocytes and 10% ESCs in the autologous EBCs. As the most important component of EBCs, the ESCs have a high capacity of self-renewal and generate daughter cells that move up from the basal layer and undergo gradually differentiation to epidermal [7]. In the past 2 years, how to make full use of the ESCs in the autologous EBCs to improve its therapeutic effect has been our major project.

FN is a multifunctional protein most abundant in the ECM under dynamic remodeling conditions, especially in wound healing [8, 9]. With its fibrillar structure, FN reinforces perimatrix formation, where it not only serves as a biological glue mediating the interaction between cells and other ECM proteins, but also serves as a pericellular structure for collagen matrix deposition which was degraded in the remodeling phase by activated tissue cells, including epidermal cells, fibroblasts, and endothelial cells [10, 11]. Since Jones PH and Watt FM found epidermal stem cells could be attached to the FN-coated plates faster than other skin cells, it has been used in many in vitro studies to obtain stem cells [12]. What is more, FN has also been proven to have a strong ability to promote cell proliferation and migration [10, 13, 14]. In recent years, FN has been proven to play an anti-infection role by regulating the monocyte-macrophage system to remove residual bacteria from the wound and has been used on wound management [15]. Taken together, we believe that FN precoating wound bed before EBCs spray can significantly improve the cell utilization and therapeutic effect.

The present study was conducted in vitro and in vivo to investigate whether precoating FN on the wound bed before spraying the autologous EBCs could enhance the full-thickness wounds healing by improving the ESCs utilization and detect its optimal concentration and possible mechanism. The results of our study not only discovered an optimal concentration for FN precoating and its excellent effects on full-thickness wounds but also demonstrated an exciting new way that may help us to explore more innovative approaches for full-thickness wound healing.

## Methods

### Ethics statement

Human EBCs were isolated from the donor skin (1 × 1 cm^2^) of patients who needed skin grafting from the Department of Burn Surgery, The First Affiliated Hospital of Sun Yat-sen University. The informed consent was obtained from the volunteers or their legal representatives prior to their participation. The research procedure was performed in compliance with the Declaration of Helsinki and approved by the Institutional Review Board of First Affiliated Hospital of Sun Yat-sen University.

All animal experiments were approved by the Institutional Animal Care and Use Committee at Sun Yat-Sen University and were conducted under the National Institutes of Health guidelines. SD rats (male, age 6–7 weeks, weight 200–250 g, grade SPF) were obtained from the Experimental Animal Center of Sun Yat-Sen University (license: SYXK 2017-0081) and housed under standard laboratory conditions according to the regulation of ethics committee of the Medical Sciences Department.

### Isolation of EBCs and ESCs

Human EBCs were isolated from the donor skin (1 × 1 cm^2^) of patients who needed skin grafting, as our previous publications [5]. Briefly, the skin was obtained with a Zimmer® dermatome (Zimmer, Dover, Ohio, USA) and ReCell® kit (Avita Medical, Cambridge, UK) and cut into strips (approximately 0.5 × 1 cm^2^). After being incubated in 1 mg/ml Dispase (17105041; Gibco) in DPBS with epidermal facing up at 4 °C overnight, the epidermal sheets were carefully separated from the dermis and minced into pieces (0.1 × 0.1 cm^2^). The small pieces of epidermis were transferred to a 50-ml centrifuge tube and digested in 0.25% trypsin (25200-056; Gibco) constantly shaken at room temperature for 15 min. Twenty-milliliter Dulbecco’s modified Eagle’s medium (DMEM; 12100-046; Gibco) containing 10% FBS was added to inactivate the trypsin. Followed by filtered and centrifuged, the EBCs were resuspended in keratinocyte serum-free medium (K-SFM; 17005042; Gibco).

To isolate the ESCs, the resuspended EBCs were seeded at a density of 10^5^ cells/cm^2^ in flasks coated with 100 μg/ml collagen IV (C-6745; Sigma, USA) for 20 min or 5–40 μg/ml FN (Shanghai Fibronectin Biotechnology, Shanghai, China) for 10 min at 37 °C. The rapidly adhering cells were considered to be ESCs. Then, the unadhered cells were removed by changing medium and the adhered ESCs were cultured in K-SFM medium at 37 °C in 5% CO2. When the culture reached 70–80% confluence, the cells were digested and passaged at a ratio of 1:2 [16]. Meanwhile, the cells were identified to be ESCs with integrin-α6^bri^ (3750S; CST, USA) and CD71^dim^ (553,264; BD) by immunofluorescence staining.

The EBCs and ESCs of rats were isolated from the dorsal skin of SD rats as our previous report with the similar procedure of Human’s [16].

### Cell treatment

To investigate the effect of FN and collagen IV coating on ESCs, the cell experiments were divided into six groups, which were planted on the culture dishes precoated with 5, 10, 20, and 40 μg/ml FN and 100 μg/ml collagen IV respectively. Control group were cultured in KSFM alone and added 1 ml DPBS. The adherent and colony formation rate, proliferation, and migration of ESCs were compared sequentially.

### Cell adhesion assay

To detect the ability of FN and collagen IV to adhere ESCs from EBCs, approximately 10^6^ autologous EBCs were seeded into the 35-mm cell culture dishes precoated with FN (5, 10, 20, or 40 μg/ml) and collagen IV (100 μg/ml). Each concentration group contained 3 dishes and washed twice after 10 min (for FN-coated dishes) or 20 min (for collagen IV-coated dishes) to remove the non-adherent cells [12]. After being photographed by microscope, the attached cells were digested for counting and calculating the adhesion rate.

### Colony formation assay

1 × 10^3^ ESCs were seeded into 35-mm cell culture dishes precoated with FN (5, 10, 20, or 40 μg/ml) and collagen IV (100 μg/ml) and cultured in KSFM for 10 days. The medium was removed at day 10; the cells were washed in DPBS, fixed in 4% paraformaldehyde, and stained with 0.5% crystal violet for 20 min. The stained cell colonies were washed with DPBS three times, air dried, and photographed. Images were obtained by a digital camera, and colonies were counted using ImageJ software. The colony-forming efficiency (CFE) was calculated by the following formula: (number of colonies formed/number of cells inoculated) × 100.

### Cell proliferation assay

The effects of FN coating on the proliferation of ESCs were evaluated by the Cell Counting Kit-8 (CCK-8; Dojindo Molecular Technologies, USA) according to the manufacturer’s instructions. The CCK-8 assay is based on the dehydrogenase activity detection in viable cells. Its main ingredients WST-8 produce a water-soluble formazan dye by dehydrogenases in viable cells, which is directly proportional to the number of living cells. Six identical 96-well plates precoated with different concentrations of FN and collagen IV were prepared for 6 time points. ESCs were seeded into the plates at 1 × 10^3^ cells/well, and each group was assessed in triplicate. Ten-microliter CCK-8 solution was added into culture medium at days 0, 1, 2, 3, 4, and 5, respectively, and incubated at 37 °C in a 5% CO2 for 2 h. Optical density (OD) value was measured at 450 nm by a microplate Spectrophotometer (Epoch, BioTek Instruments, USA).

### Cell migration assays

For cell migration assays, 3 × 10^5^ ESCs were seeded into 6-well plates precoated with 20 μg/ml FN and 100 μg/ml collagen IV respectively and grown to a confluence of 80–90% in conventional scratch-wound assays. Ten micrograms per milliliter mitomycin C was added into the medium 6 h before scratching to inhibit cell proliferation. One-milliliter sterile pipette tips were used to make uniform linear scratches on cell monolayer. The cell debris was washed gently with KSFM, and images were immediately captured using an inverted microscope equipped with a digital color camera (XC30, Olympus Inc.). The exact location of the image was marked to identify the same gap over the next 24 h. Images were taken in a timely manner and measured by Image-Pro Plus 6.0 software.

### Animal study

To explore the function of FN precoating on full-thickness wound bed in vivo, the rats’ dorsal wound model was adopted [17]. Ninety rats were anesthetized by inhaling isoflurane (INH), and a diameter of 2-cm full-thickness wound was made on the dorsal skin of each rat. The wounds were divided into three groups randomly: control group (PBS), EBCs group, and FN + EBCs group. The EBCs were isolated from the excised dorsal skin as the procedure we mentioned above and evenly spray to the wound bed by a 2-ml syringe. For the FN + EBCs group, 20 μg/ml FN were evenly precoating on the wound bed for 30 min by a 2-ml syringe before EBC spray (Fig. 3a). The rats and wounds were observed, photographed, and measured daily until the rats were sacrificed. Wound healing time was recorded, and the residual wound area rate was calculated as [(day *n* area)/(day 0 area)] × 100% (*n* = 0, 3, 7, 14 or 21). Six rats of each group were sacrificed at days 0, 3, 7, 14, and 21, respectively, and the wound tissues were harvested and separated into two halves across the center: one half was processed for histological and immunohistochemistry analysis, and the other was rapidly frozen in liquid nitrogen for RNA and protein analysis.

### Histological and immunohistochemistry staining analysis

For histological study, skin tissue samples were routinely fixed with 10% formalin and embedded in paraffin. The 4-μm paraffin sections of each group at days 3, 7, 14, and 21 were deparaffinized and stained with hematoxylin and eosin (H&E) and were examined with standard light microscopy (Olympus, Japan) to observe the skin epidermis, dermis, accessories, inflammation, and collagen deposition.

For immunohistochemistry staining, the paraffin-embedded fixed tissue sections of each group were deparaffinized and rehydrated in xylene and graded ethanol. Antigen retrieval was performed using Proteinase K solution (20 μg/ml) at 37 °C for 15 min. Following Bloxall blocking, the sections were blocked with mouse serum for 30 min and then incubated with primary mouse monoclonal anti-rat antibodies: anti-CD31 (1:100, ab64543; Abcam) overnight at 4 °C in a humidified container. After washing in PBST, the sections were incubated with an HRP conjugated secondary antibody (1:2000, ab97051; Abcam) for 1 h at room temperature. The sections were further incubated with 3,3′-diaminobenzidine (DAB) and counterstained with hematoxylin and observed by microscope.

### Immunofluorescence analysis

Formalin-fixed and paraffin-embedded tissue sections were deparaffinized in xylene and rehydrated through graded ethanol. Antigen retrieval was performed using citrate buffer in a pressure cooker at 95 °C for 30 min. The 4-μm sections of each group were blocked in 10% goat serum (16210064; Gibco) for 30 min at 37 °C. For double labeling, two compatible primary anti-rats antibodies were added: anti-K15 (1:250, ab80522; Abcam) and anti-Ki-67 (1:200, ab16667; Abcam). After incubating at 4 °C overnight, the sections were washed with PBST and incubated with the following secondary antibodies for 1 h: goat anti-rabbit IgG labeled with Alex Fluor 488 (1:200, ab150077; Abcam) and goat anti-mouse IgG labeled with Alexa Fluor 594 (1:200, ab150116; Abcam). Sections were documented with a fluorescence microscope (OLYMPUS, Japan).

### RNA extraction and quantitative real-time reverse transcription PCR (qRT-PCR) analysis

Total RNA was isolated from the skin sample of rats by Trizol reagent (Invitrogen, CA, USA) and transcribed into cDNA using the PrimeScript RT reagent Kit (TaKaRa, Dalian, China) following the manufacturer’s protocol. The qRT-PCR reactions were performed with SYBR Premix ExTaq (TaKaRa, Dalian, China) and detected by the Mx3000P real-time PCR system (Stratagene, Agilent Technologies, CA, USA). All primer sequences used for mRNA expression analysis are shown in Table 1. Melting curves were generated to verify the specificity of each qRT-PCR reaction. The GAPDH gene was used as a reference gene for mRNA quantification. The relative expression ratio of mRNA was calculated by the 2^−ΔΔCT^ methods. An independent experiment was repeated three times for accuracy.

**Table 1:** Primer sequences for quantitative real-time reverse transcription PCR

Gene	Direction	Sequence 5′ → 3′
Tumor necrosis factor-α (TNF-α)	Forward	CATCCGTTCTCTACCCAGCC
	Reverse	AATTCTGAGCCCGGAGTTGG
Interleukin-8 (IL-8)	Forward	TCCACTCCCAGCATCGTAGA
	Reverse	CAGAGTAAAGGGCGGGTCAG
Interleukin-10 (IL-10)	Forward	CTTCGGCCCAGTGAAGAGTT
	Reverse	TGGAGCTTGCTAAAGGCACT
MKI 67 (Ki-67)	Forward	GGAGGGTTTCCAGACACCAG
	Reverse	ACTTGCCCTAGACTGTCTCTCT
Keratin 15 (K15)	Forward	GCTTGCTAGGGACCCGAAG
	Reverse	CTCGGGTAGAGCCACTTCCA
Cluster of differentiation 31 (CD31)	Forward	CTCCATCCTGTCGGGTAACG
	Reverse	TTCTTCGTGGAAGGGTCTGC
Matrix metallopeptidase 9 (MMP-9)	Forward	CGCCAACTATGACCAGGATA
	Reverse	GTTGCCCCCCCAGTTACAGT
Collagen I (Col I)	Forward	GTACATCAGCCCAAACCCCA
	Reverse	CAGGATCGGAACCTTCGCTT
Collagen III (Col III)	Forward	ATATGTGTCTGCGACTCGGG
	Reverse	GGGCAGTCTAGTGGCTCATC
Glyceraldehyde-3-phosphate dehydrogenase (GAPDH)	Forward	AGACAGCCGCATCTTCTTGT
	Reverse	TGATGGCAACAATGTCCACT

### Protein extraction and western blot analysis

Western blot was carried out as described previously. The rats’ skin sample from the wound area was used to detect the inflammation and collagen deposition. Total proteins of rats’ skin samples were extracted with the ProteoPrep® Total Protein Extraction Kit (Sigma, USA), and its concentration was determined by the Bicinchoninic acid assay kit (BCA, 23225; Pierce). Equal amounts of protein extract (30 μg) were subjected to electrophoresis in 10% SDS-PAGE gels at 100 V for 2 h and then transferred to PVDF membranes (Millipore, USA) at 100 V for 90 min. After blocking with 5% nonfat milk in a mixture of Tris-buffered saline solution and Tween-20 (TBST, 0.1 M, pH 7.4) for 1 h at room temperature, membranes were incubated overnight at 4 °C with one of the following primary rabbit anti-rats antibodies: anti-MMP-9 (1:1000, ab38898; Abcam), anti-Collagen I (1:1000, ab34710; Abcam), anti-Collagen III (1:1000, ab7778; Abcam), anti-TNF-α (1:1000, ab6671; Abcam), anti-IL-8 (1:10, ab7747; Abcam), and anti-IL-10 (1:1000, ab9969; Abcam). Afterward, the membranes were incubated with peroxidase-conjugated secondary antibody IgG (1:2000, ab6721; Abcam) for 1 h at room temperature. The immunoreactive bands were visualized by the enhanced chemiluminescent detection system (ECL, Amersham) and analyzed by Image Pro-Plus 6.0 software (Media Cybernetics). Quantitative analysis of the target protein bands was normalized by corresponding measures of GAPDH derived from the same samples. Each western blot analysis was run in three independent replicates.

### Statistical analysis

Data were analyzed with PRISM 7.0 software (GraphPad, CA, USA). Values were expressed as the mean ± standard deviation (SD) unless otherwise indicated. Comparisons of expression difference between control and experimental groups were conducted by Student’s *t* test. The differences between multiple groups were compared using one-way analysis of variance (ANOVA), followed by a Bonferroni post hoc test for pairwise comparisons. All statistical analyses were performed by SPSS 19.0 software (SPSS, Chicago, IL, USA), and *P* < 0.05 indicates that the difference was statistically significant.

## Results

### FN precoating culture dishes accelerate the adhesion, proliferation, CFE, and migration of ESCs in vitro

**Fig. 1:**
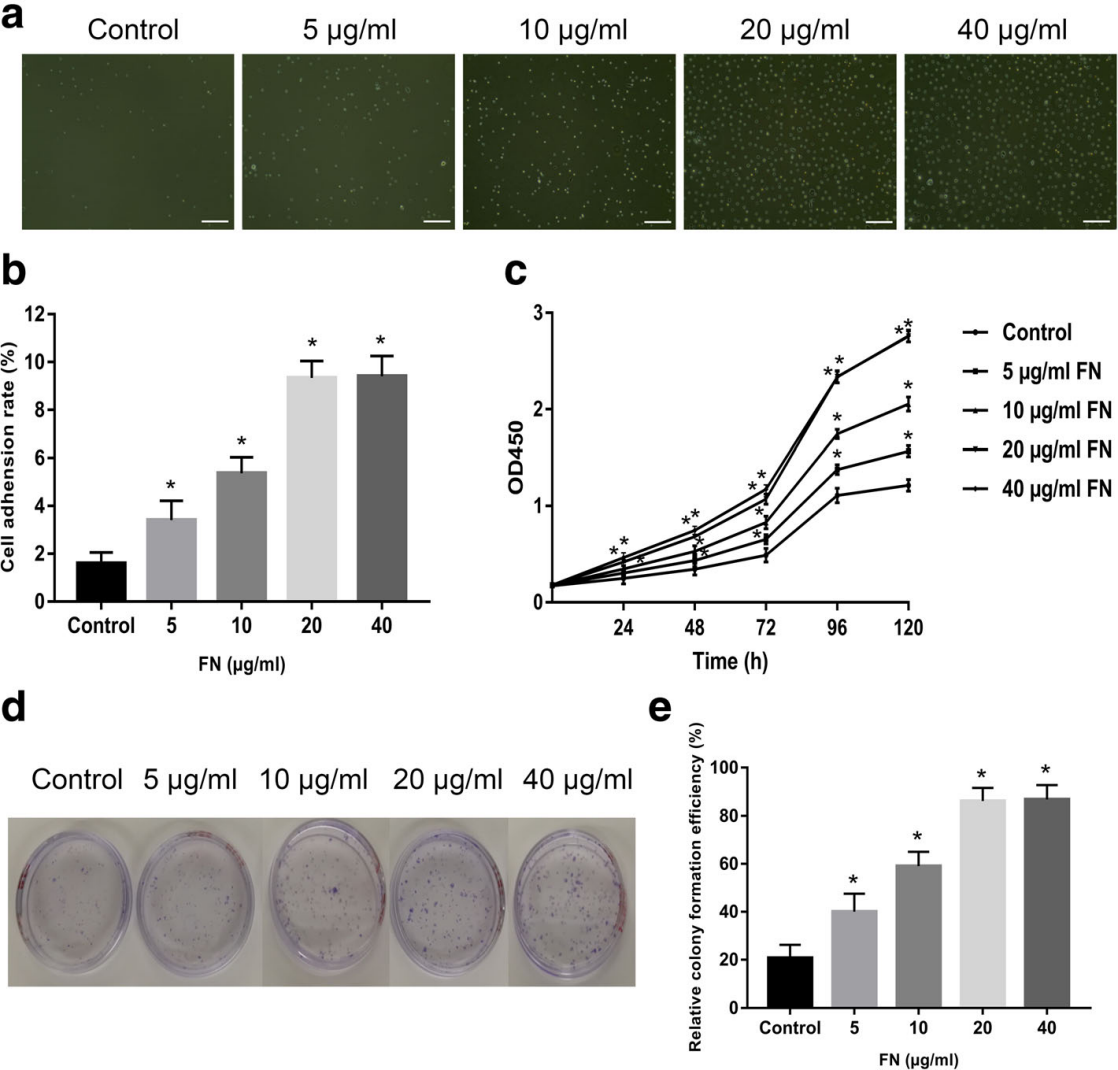
The effects of FN precoating on ESCs’ adhesion, proliferation, and colony formation. **a**, **b** Representative pictures of adherent ESCs in different concentrations’ FN-precoated dishes and their adhesion rates. **c** The proliferation of ESCs in different concentrations’ FN-precoated dishes was analyzed by CCK-8 assay. **d**, **e** Representative picture of ESCs’ colony formation and their cloning efficiency in different concentrations FN-precoated dishes. The values were analyzed by Graph Prism 7.0. Error bars represent SEM. Student’s *t* test, *P < 0.05 compared with control value (*n* = 5). Scale bar, 200 μm

To detect the effects of FN precoating on ESCs’ adhesion, different concentrations of FN were used to precoat cell culture dishes. After being incubated in 37 °C for 10 min, the attached cells were photographed by a microscope (Fig. 1a). Then, they were digested for counting and calculating the adhesion rate (Fig. 1b). As shown in Fig. 1, the cell adherence rate was increased in a dose-independent manner with the concentration of FN. However, when the concentration of FN reached 20 μg/ml, this function tends to be stable. Analogously, the CCK-8 analysis (Fig. 1c) and CFE assay (Fig. 1d, e) also depicted that 20 μg/ml could be the best concentration for ESCs’ proliferation.

**Fig. 2:**
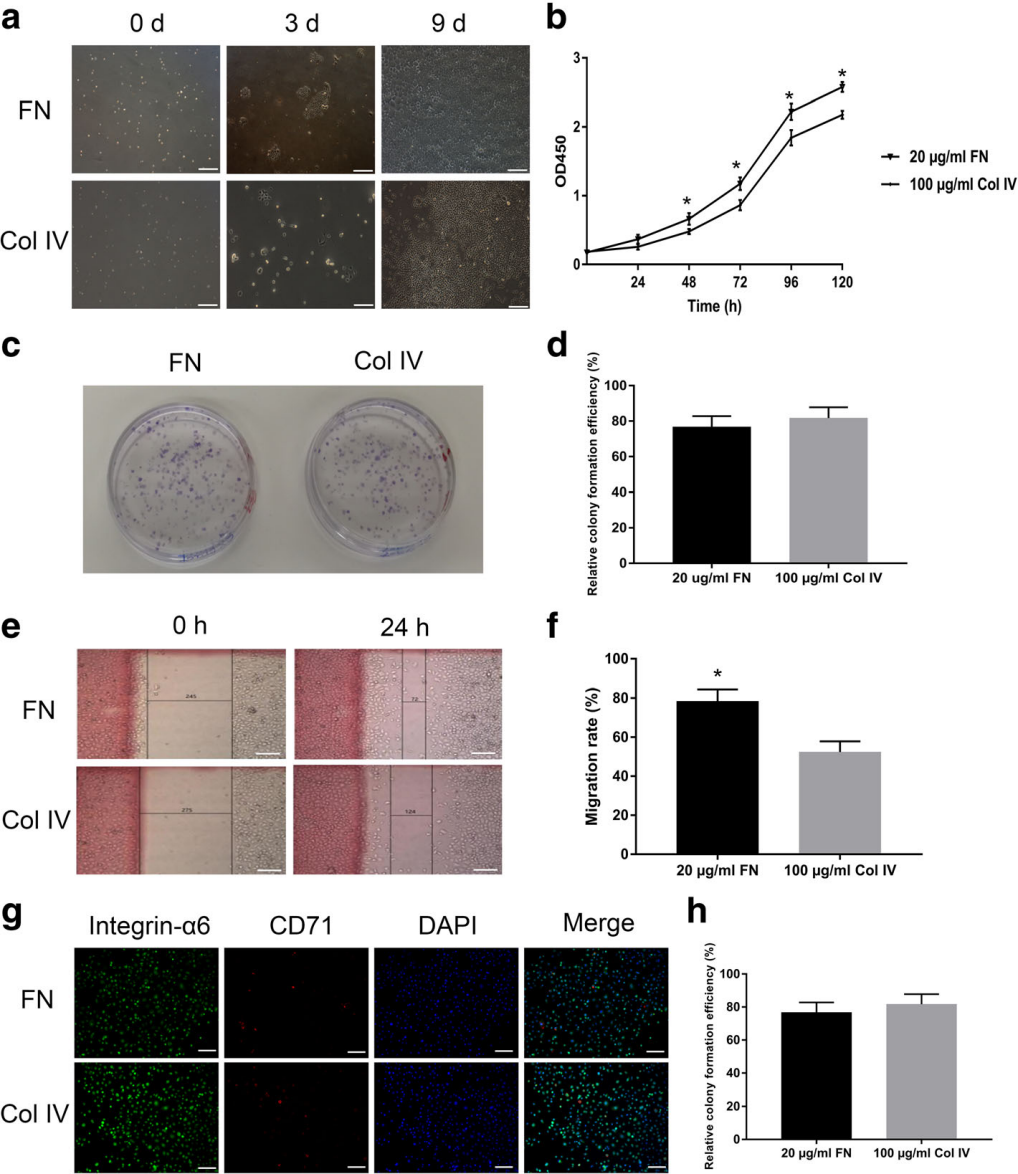
The effects of 20 μg/ml FN and 100 μg/ml collagen IV precoating on ESCs’ adhesion, proliferation, and colony formation. **a** Representative pictures of ESCs’ adhesion and proliferation in 20 μg/ml FN- and 100 μg/ml collagen IV-precoated dishes. **b** The proliferation of ESCs in 20 μg/ml FN- and 100 μg/ml collagen IV-precoated dishes were analyzed by CCK-8. **c**, **d** Representative pictures of ESCs’ colony formation and their cloning efficiency in 20 μg/ml FN- and 100 μg/ml collagen IV-precoated dishes. **e**, **f** Representative pictures of ESCs’ scratch-wound assays and their migration rate in 20 μg/ml FN- and 100 μg/ml collagen IV-precoated dishes. **g**, **h** Representative pictures of ESCs’ purity and their rates among all the primary adherent cells in 20 μg/ml FN- and 100 μg/ml collagen IV-precoated dishes. The values were analyzed by Graph Prism 7.0. Error bars represent SEM. Student’s *t* test, *P < 0.05 compared with control value (*n* = 5). Scale bar, 200 μm

As 100 μg/ml collagen IV-precoated dishes have been used to rapidly sort ESCs from EBCs for many years [18], we compared the effects on the ESCs’ adhesion, proliferation, migration, and CFE between 20 μg/ml FN- and 100 μg/ml collagen IV-precoated dishes in this study. As shown in Fig. 2, there was no significant difference in ESCs’ adhesion and CFE (Fig. 2a–d), but the proliferation and migration of ESCs in the FN precoating group were obviously enhanced than in the collagen IV precoating group (Fig. 2a, b, e, f). To identify the ESCs proportion in the rapid adhesive cells, the integrin-α6 and CD71 were detected by a fluorescence microscope, and the results showed there was no significant difference in ESCs’ purity between the FN and collagen IV precoating groups (Fig. 2g, h).

### FN precoating wound bed improves wound closure and healing quality of SD rats

**Fig. 3:**
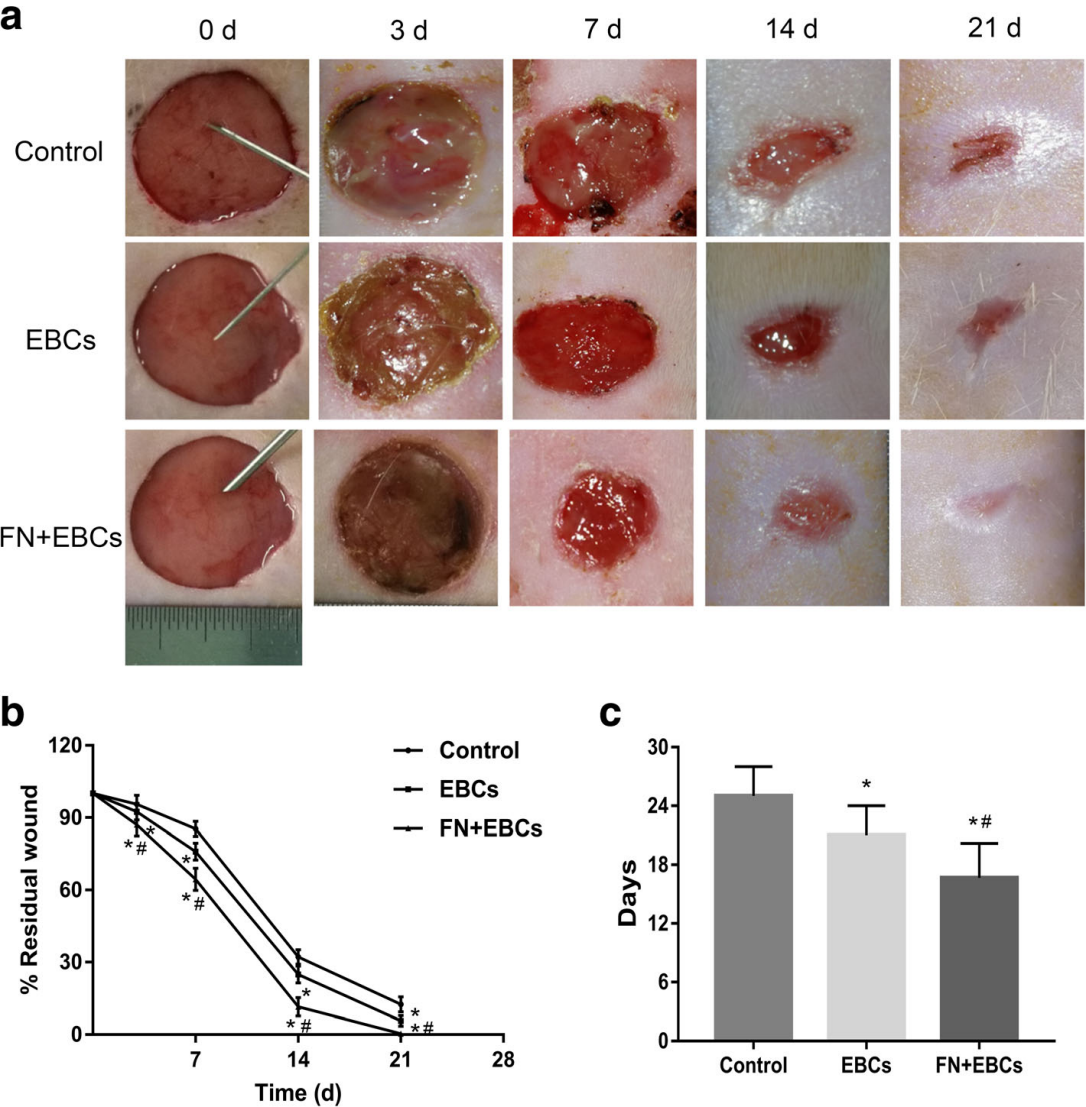
FN precoating wound bed accelerated wound closure and improved healing quality of SD rats. Full-thickness dermal wounds were created on the dorsal skin of SD rats and treated by PBS (control), EBCs, and FN + EBCs respectively. **a** Representative wound pictures of rats’ dorsal from each group taken on post-injury days 0, 3, 7, 14, and 21. **b**, **c** Residual wound rates and completed wound healing time of each group. The computation was that the indicated area was divided by the initial area. Results represent means ± SEM. The values were analyzed by Graph Prism 7.0. Error bars represent SEM. Student’s *t* test, *P < 0.05 compared with control value (*n* = 6), ^#^*P* < 0.05 compared with EBCs value (*n* = 6)

To investigate the role of FN precoating wound bed in vivo, we implemented our experiments using the rats’ dorsal wound model. As Fig. 3 displayed, compared with the control group, the EBCs group, and the FN + EBCs group showed a dramatically higher healing quality (Fig. 3a), lower residual wound area (Fig. 3b), and shorter healing time (Fig. 3c). What is more, compared with the EBCs group, the FN + EBCs group showed an obviously higher healing quality (Fig. 3a), lower residual wound area (Fig. 3b), and shorter healing time (Fig. 3c). These results suggested that FN precoating wound bed before EBCs spray could promote wound healing and improve the healing quality significantly.

### FN precoating wound bed promotes re-epithelialization and decreases inflammation

**Fig. 4:**
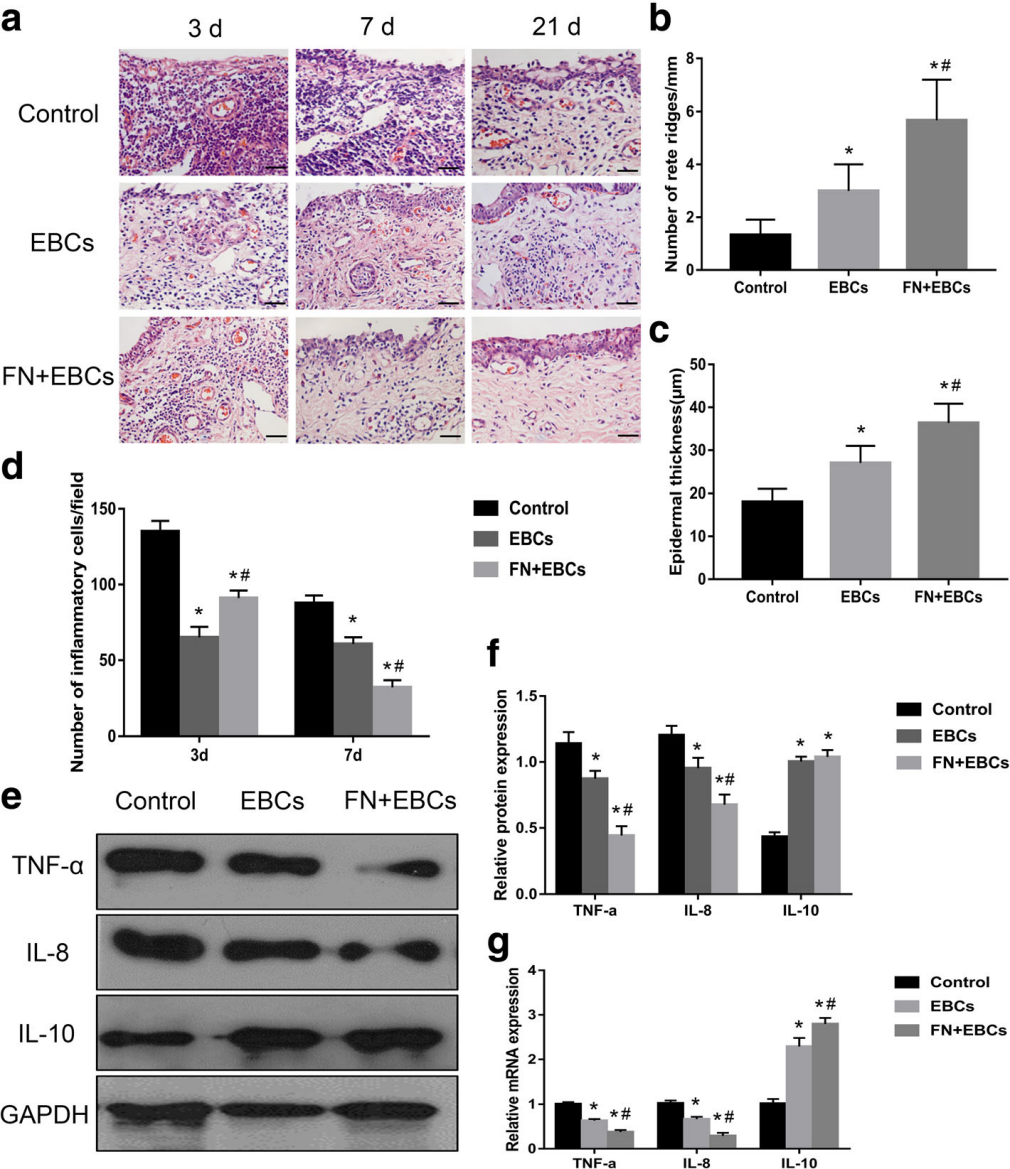
Histological features and expression of inflammatory factors of the rats’ dorsal wounds in each group. **a** Wound tissue sections stained with H&E on post-injury days 3, 7, and 21 showing histological features in rats’ dorsal wound treated with PBS (control), EBCs, and FN + EBCs. **b**, **c** Analyses of rate ridges numbers and epidermal thickness of wound tissue sections treated with PBS (control), EBCs, and FN + EBCs on post-injury day 21 showing histological features in rats’ dorsal wound. FN + EBCs treated wound displayed significantly more rate ridges and thicker epidermis than the others. **d** The number of inflammatory cells on day 3 and day 7 was quantified at per × 40 magnification for five areas randomly, FN + EBCs treated wound displayed significantly lower inflammatory response and fewer inflammatory cells on day 7. **e**, **f** Representative western blot and results of densitometric analysis of blots showing relative protein levels of TNF-α, IL-8, and IL-10 for each group on post-injury day 7. **g** Representative qRT-PCR analysis showing relative mRNA levels of TNF-α, IL-8, and IL-10 for each group on post-injury day 7. The values were analyzed by Graph Prism 7.0. Error bars represent SEM. Student’s *t* test, *P < 0.05 compared with control value (*n* = 6), ^#^*P* < 0.05 compared with EBCs value (*n* = 6). Scale bar, 100 μm

To further evaluate the wound healing quality, we collected the wound area tissue specimen at specific time points (days 3, 7, and 21) and observed re-epithelialization by H&E staining. As shown in Fig. 4, the wound healing quality of the FN + EBCs group and the EBCs group were significantly better than the control group (Fig. 4a), with more epidermal ridges (Fig. 4b) and thicker epidermis (Fig. 4c). However, when the wound bed was precoated by FN before EBCs spraying, the re-epithelialization, epidermal ridges, and epidermal thickness were significantly improved (*P* < 0.05). These results suggested that FN precoating wound bed had an active function in promoting re-epithelialization and improving wound healing quality, which might mainly be by increasing the ESCs utilization of EBCs suspension.

To detect the inflammation of the wound area, we collected the samples on day 3 and day 7. By H&E staining (Fig. 4a), we found the number of inflammatory cells of the FN + EBCs group and the EBCs group was much less than the control group at both time points (Fig. 4d). However, when comparing the FN + EBCs group with the EBCs group, we discovered that the wound precoated with FN displayed more inflammatory cells on day 3 but less inflammatory cells on day 7 (Fig. 4d). Furthermore, we detected the inflammatory factor of wound tissue snap-frozen samples on day 7 by western blot and qRT-PCR, and the results showed the FN + EBCs group displayed significantly decreased in TNF-α and IL-8 expression, while increased in IL-10 expression (Fig. 4e–g). These results demonstrated FN precoating wound bed before EBCs spray could enhance the inflammatory response at the early stage while reducing the inflammation as the wound healing is going on.

### FN precoating wound bed promotes the adhesion and proliferation of ESCs

**Fig. 5:**
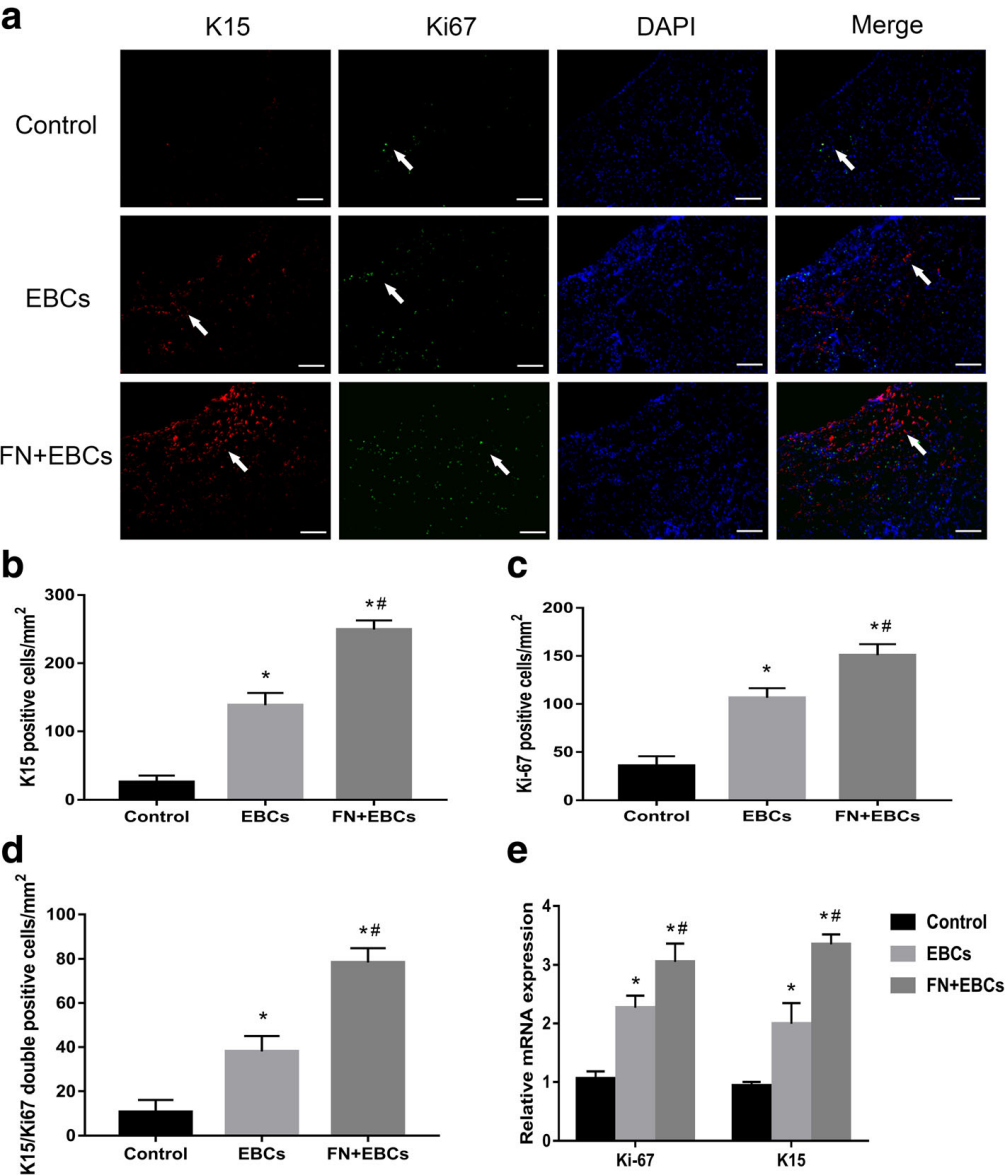
FN precoating wound bed promotes the adhesion and proliferation of ESCs. **a**–**c** Represent area and analysis of wound tissue sections stained with K15 and Ki-67 on post-injury day 7 showing the number of ESCs and proliferating cells in rats’ dorsal wound treated with PBS (control), EBCs, and FN + EBCs. Arrows indicate the positive cells. **d** Analysis of wound tissue sections double-stained with K15 and Ki-67 on post-injury day 7 showing the number of growing ESCs in rats’ dorsal wound treated with PBS (control), EBCs, and FN + EBCs. **e** Representative qRT-PCR analysis showing relative mRNA levels of Ki-67 and K15 for each group on day 7. The values were analyzed by Graph Prism 7.0. Error bars represent SEM. Student’s *t* test, *P < 0.05 compared with control value (*n* = 6), ^#^*P* < 0.05 compared with EBCs value (*n* = 6)

To further confirm the mechanism of FN precoating wound bed before EBCs spray, the expression of K15 and Ki-67 of wound area tissue sections on day 7 were detected by immunofluorescence. As shown in Fig. 5, the K15-positive, Ki-67-positive, and K15/Ki-67 double-positive cells in the FN + EBCs group were significantly more than those in the EBCs group and the control group (Fig. 5a–d). Then, we further discovered their mRNA expression of wound tissue snap-frozen samples on day 7 by qRT-PCR and found the mRNA expression of K15 and Ki-67 was dramatically higher in the FN + EBCs group compared with the EBCs group and the control group (Fig. 5e). These results revealed FN precoating wound bed could promote ESCs attached to the wound bed and increase the utilization and proliferation of ESCs and other cells which exists in the wound bed.

### FN precoating wound bed promotes angiogenesis

**Fig. 6:**
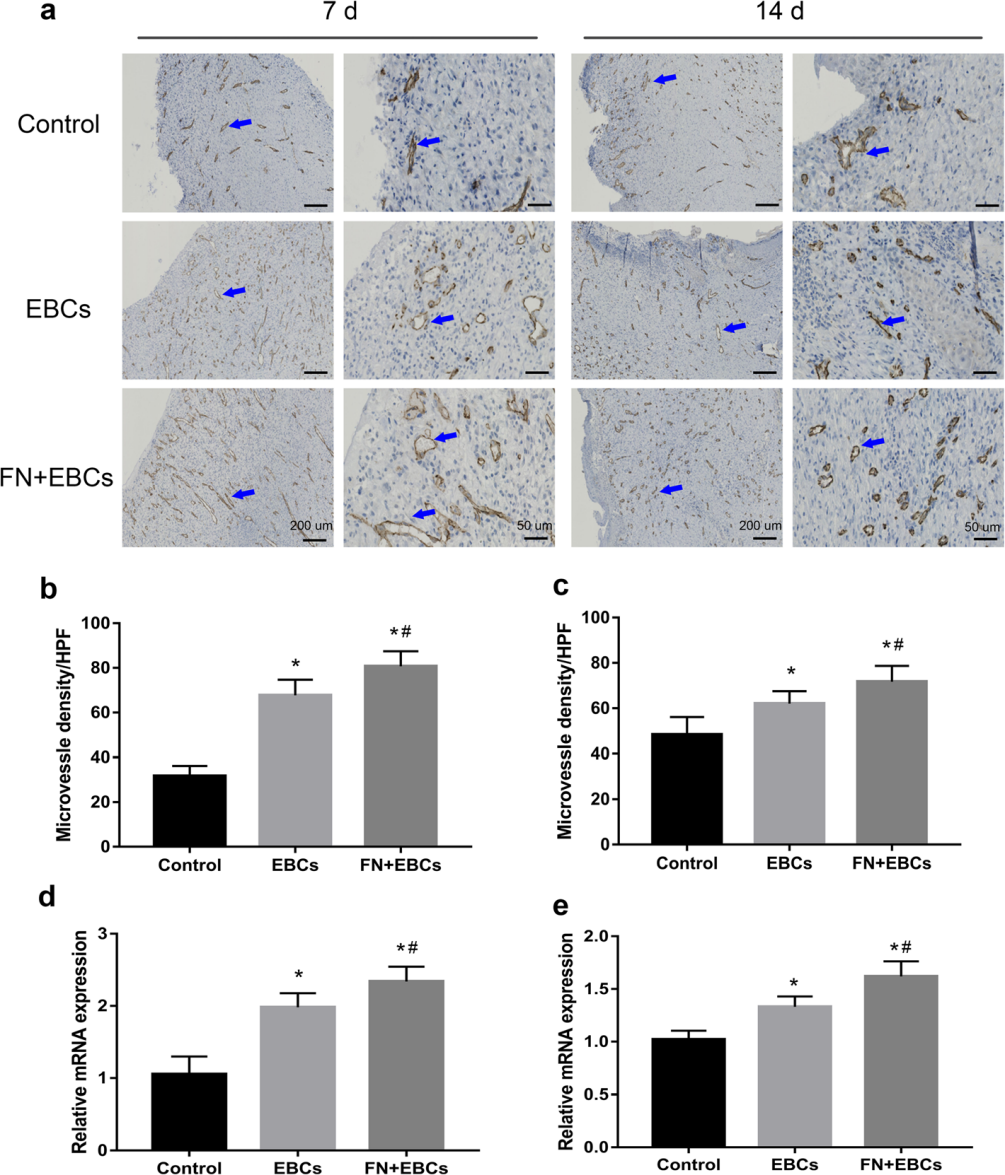
The expression of angiogenesis factors of the rats’ dorsal wounds on day 7 and day 14 in each group was analyzed by IHC. **a**–**c** Represent area and analysis of wound tissue sections stained with CD31 on post-injury day 7 and day 14 showing the microvascular regeneration in rats’ dorsal wound treated with PBS (control), EBCs, and FN + EBCs. Arrows indicate the positive cells. **d**, **e** Representative qRT-PCR analysis showing relative mRNA levels of CD31 for each group at day 7 and day 14. The values were analyzed by Graph Prism 7.0. Error bars represent SEM. Student’s *t* test, *P < 0.05 compared with control value (*n* = 6), ^#^*P* < 0.05 compared with EBCs value (*n* = 6)

To assess the angiogenesis, the wound area sections on day 7 and day 14 were stained with CD31 for immunohistochemistry. Microvessel density (MVD) was assessed through CD31-positive cells at per × 40 magnification for five areas randomly. As shown in Fig. 6, the FN + EBCs group displayed significantly higher MVD than the EBCs group and the control group at both time point, and the CD31 expression was strong positive (Fig. 6a–c). Similarly, the qRT-PCR of the wound snap-frozen samples on day 7 and day 14 also showed a markedly higher CD31 mRNA expression in FN precoating group compared with the others (Fig. 6d, e), which determined that FN precoating wound bed could improve wound healing by accelerating angiogenesis.

### FN precoating wound bed regulates the collagen formation and degradation

**Fig. 7:**
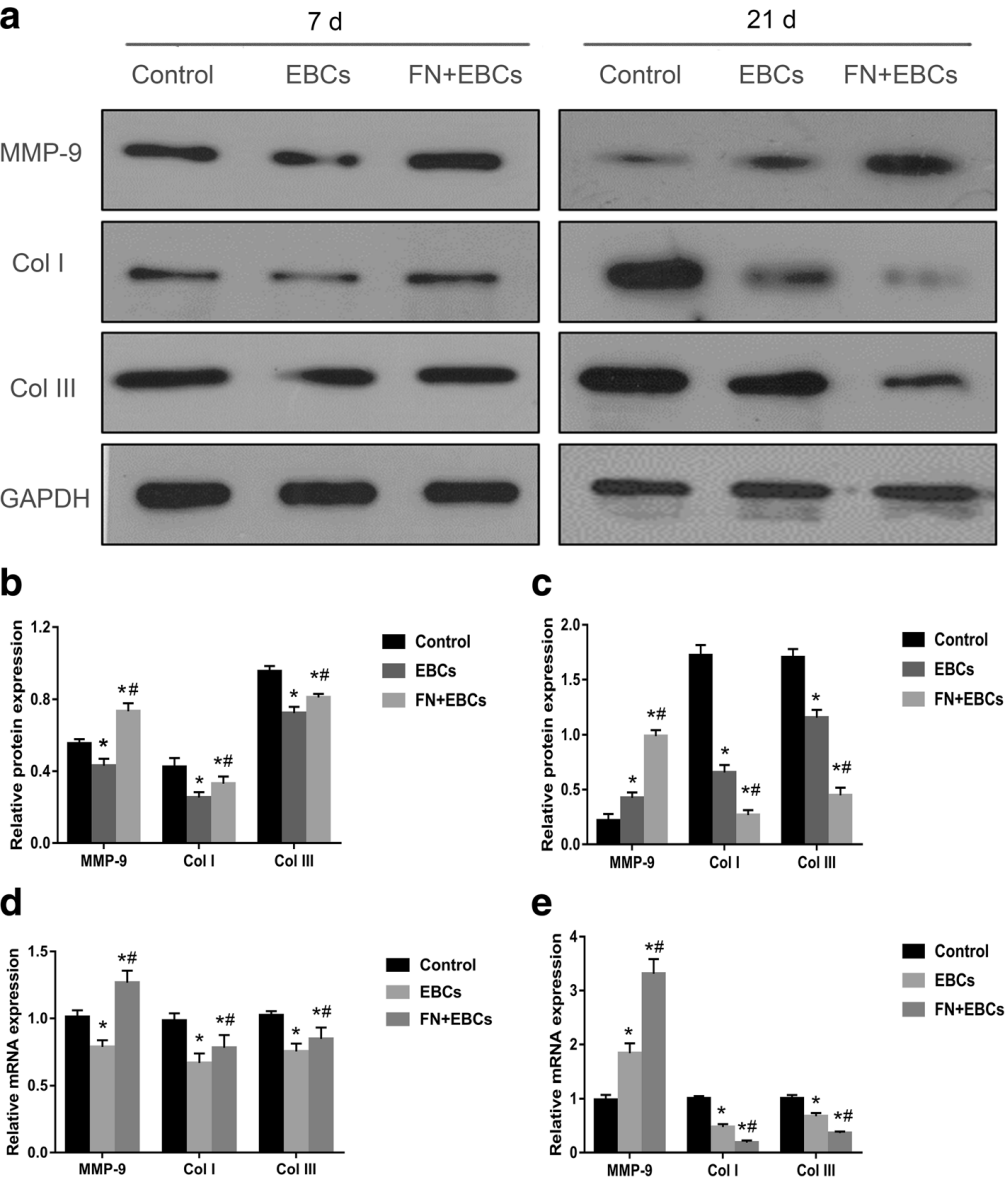
FN precoating wound bed regulates the collagen formation and degradation. **a**–**c** Representative immunoblot and results of densitometric analysis of blots showing relative protein levels of MMP-9, Col I, and Col III for each group on post-injury day 7 and day 21. **d**, **e** Representative qRT-PCR analysis showing relative mRNA levels of MMP-9, Col I, and Col III for each group on post-injury day 7 and day 21. The values were analyzed by Graph Prism 7.0. Error bars represent SEM. Student’s *t* test, *P < 0.05 compared with control value (*n* = 6), ^#^*P* < 0.05 compared with EBCs value (*n* = 6)

To detect the effects of FN precoating wound bed on collagen formation and degradation, the wound samples collected from day 7 and day 21 were analyzed by Western Blot and RT-qPCR. As shown in Fig. 7, on day 7, compared with the control group, the expression of collagen I and collagen III were downregulated in the FN + EBCs group and the EBCs group, while the expression of MMP-9 was upregulated in the FN + EBCs group but downregulated in the EBCs group (Fig. 7a, b, d). However, when compared with the EBCs group and the control group on day 21, the expression of collagen I and collagen III in the FN + EBCs group were dramatically decreased while the expression of MMP-9 was significantly increased (Fig. 7a, c, e). These results revealed that FN precoating wound bed could promote wound healing by accelerating the collagen formation at the early stage, while improving wound healing quality by promoting collagen degradation as wound healing is going on.

## Discussion

As a common and frequently occurring disease, full-thickness wounds have been plaguing the patients and their doctors for many years, but still lack effective treatment. Although autologous EBCs therapy has greatly improved the treatment effects of the full-thickness wounds, we found it remained three major deficiencies that seriously affected the utilization of EBCs. In our present study, we demonstrated FN precoating wound bed before EBCs spray could defeat these deficiencies and improve its utilization and function, especially the ESCs in it. We first detected the effects of FN precoating culture dishes on ESCs in vitro, found 20 μg/ml was the optimal concentration for FN coating, and then verified our results on rats’ dorsal wound model. Our results revealed that FN precoating wound bed before EBCs spray could significantly promote full-thickness wound healing by improving the utilization and function of the ESCs and further promoting angiogenesis, decreasing inflammations, and regulating collagen formation and degradation.

As the main deficiency of current autologous EBCs therapy was the low utilization of ESCs, it is important to adhere ESCs more efficiently. Since Jones and Watt found ESCs could be attached to FN coating flask in 10 min at 37 °C, FN has been used to sort ESCs from EBCs for a long time, but the optimal concentration of FN remains unknown [12]. In our present study, we detected different concentrations of FN precoating culture dishes on the effects of attachment rate, colony formation rate, proliferation, and migration of ESCs and found the 20 μg/ml FN was the optimal concentration. As 100 μg/ml collagen IV has been widely used for sorting ESCs from EBCs [18], we compared the effects on ESCs between 20 μg/ml FN and 100 μg/ml collagen IV. We discovered although they were similar in ESCs attachment and colony formation, 20 μg/ml FN could significantly promote the proliferation and migration of ESCs compared with 100 μg/ml collagen IV. This result is consistent with Saito T’s recent study which found odontoblast displayed higher proliferation ability on FN-coated substrates than on collagen-coated substrates [19]. What is more, as FN has been used on the topical wound as medicine for more than 2 years and proven to be effective and safe, it would be the optimal extracellular matrix to precoat wound bed [20].

To verify our speculation in vivo, we chose classic rats’ dorsal full-thickness wound as our full-thickness wound model. As the wound bed temperature was about 37 °C, we precoated the wound bed with 20 μg/ml FN for 30 min before EBC spray according to our in vitro results. With the support of FN, we could see the cell loss had been dramatically decreased than merely EBCs spray. This phenomenon can also be explained by Karuri NW’s research, which demonstrated that FN could serve as a biological glue mediating the interaction between cells and other ECM proteins [10]. By recording the wound healing time, measuring the wound areas, and histological staining, we found that precoating FN could significantly improve the re-epithelialization, skin attachment regeneration, and collagen reassignment, which might mainly owe to FN precoating which could accelerate the ESC attachment to wound bed and promote its proliferation and migration in site, and help the EBCs distribute more orderly.

To verify this hypothesis, we detected the K15 and Ki-67 expression of the samples on day 7. K15 has been verified as a specific marker for ESCs, and Ki-67 was used to detect the proliferation of the cells in the wound [21, 22]. As we expected, the expression of K15-positive, Ki-67-positive, and K15/Ki-67 double-positive cells was much higher in FN precoating group than the others, which revealed FN precoating wound bed could accelerate the ESC attachment to wound bed and promote its proliferation and migration dramatically. This is mainly because FN serves as a biological glue and has strong adhesive abilities for ESCs adhesion, which is consistent with our in vitro results [10].

As we all know, with the generation of blood vessel-rich granulation tissue, angiogenesis is one of the critical processes in wound healing, and many full-thickness wounds are due to ischemia [23]. To detect the angiogenesis during the wound healing, CD31 expression of samples at day 7 and day 14 were examined and analyzed by IHC to demonstrate the presence of endothelial cells [24]. We could see more blood vessels in the FN precoating group. These new blood vessels might come from the proliferation of endothelial cells and the differentiation of ESCs, which could be promoted by FN precoating [25].

Synthesized by fibroblasts, collagens impart integrity and strength to all tissues and play a key role in all phases of wound healing. The synthesis and degradation of collagens are vital for wound healing quality, especially in the proliferative and remodeling phases of repair [26, 27]. As the most important components of collagens in wound healing, collagen I and collagen III provide regenerating tissue with more strength and rigidity, but may impede cellular migration and regeneration [28]. It has been confirmed that the collagen I, collagen III, and the ratio of collagen I to collagen III increased obviously in abnormal wound healing and hypertrophic scar than scar-less wound healing [29, 30]. Our current study showed with 20 μg/ml FN precoating the wound bed, the collagen I and collagen III expression increased in the early stage, while significantly decreased after wound closure. These results revealed that FN could not only promote wound healing by increasing collagens during proliferative phase, but also improve healing qualities by degrading collagens in the remodeling phase.

To detect the mechanism of FN on the synthesis and degradation of collagens during wound healing, the timed expression and activation of Matrix metalloproteinases-9 (MMP-9) were examined and analyzed at the same time. Among the eight members of the MMPs’ family, MMP-9 is the most relevant to wound healing [31]. It has been confirmed that MMP-9 not only plays an important role in keratinocyte migration wound closure, but also is the major component in the remodeling phase of scar-free wound healing [32]. MMP-9 knockout (KO) mice display delayed wound closure and obvious scar formation highlighting the importance of MMP-9 in wound healing [33]. What is more, MMP-9 also participate in regulating angiogenesis during wound healing through the activation of proangiogenic cytokines, including TNF-a and VEGF, and by generating antiangiogenic peptides [34, 35]. Our present study showed that the expression of MMP-9 in FN precoated group was increased a little more in the proliferative phase of wound healing, while significantly higher in remodeling phase compared with the other two groups, this result revealed FN could regulate the synthesis and degradation of collagens by regulating the expression of MMP-9.

Another defect for current autologous EBCs therapy is the residual bacteria in the wound bed after debridement. These bacteria may inhibit EBCs’ proliferation and even lead a great deal of EBCs to die. It has been proven that FN has antibiotic function mainly through enhancing the infiltration ability of neutrophils and the phagocytotic activity of macrophages in wound [36]. By regulating the inflammatory response during wound healing [37], FN may create an ideal microenvironment growth for EBCs. As the most commonly used indicators for assessing inflammatory response, the increase of TNF-α and IL-8 and decrease of IL-10 indicate inflammation increasing [38, 39]. Our current study showed that the inflammatory cells in the FN precoated group were more than the EBCs group but less than the control group on day 3. However, when compared with the cell group and the control group on day 7, the inflammatory cells and proinflammatory cytokines TNF-a and IL-8 in FN precoated wound samples were dramatically decreased, while the anti-inflammatory cytokine IL-10 were significantly overexpressed. These results revealed FN precoating wound bed could regulate the inflammation during the wound closure and provide an ideal microenvironment for ESCs and then improve the wound healing eventually. In addition, despite the well-known proinflammatory effect of IL-8, it has also been shown to play an angiogenic role during wound healing [40]. However, in our present study, we found the expression of IL-8 in the FN + EBCs group was significantly lower than the control group and EBCs group on day 7, while the angiogenesis in the FN + EBCs group was much better than the others. This phenomenon indirectly indicated that FN may mainly display an anti-inflammation function during the inflammatory phase of wound healing.

## Conclusion

In summary, this work provides the first evidence that FN precoating wound bed dramatically enhances the therapeutic effects of autologous EBC therapy for full-thickness wounds by improving the utilization and function of the ESCs and further by promoting angiogenesis, decreasing inflammations, and regulating collagen formation and degradation. The results of our study not only discovered an optimal concentration for FN precoating and its excellent effects on full-thickness wounds, but also demonstrated an exciting new way which may help to inspire more innovative approaches to overcome full-thickness wounds.

## Abbreviations

BCA: Bicinchoninic acid assay kit
CCK-8: Cell counting kit-8
CD31: Cluster of differentiation 31
EBCs: Epidermal basal cells
ECL: Enhanced chemiluminescent detection system
ECM: Extracellular matrix
ESCs: Epidermal stem cells
FN: Fibronectin
GAPDH: Glyceraldehyde-3-phosphate dehydrogenase
H&E: Hematoxylin and eosin
HRP: Horseradish peroxidase
IF: Immunofluorescence
IHC: Immunohistochemistry
IL: Interleukin
K15: Keratin 1
Ki-67: MKI 67
K-SFM: Keratinocyte serum-free medium
qRT-PCR: Quantitative real-time polymerase chain reaction
SD: Sprague–Dawley
TNF-α: Tumor necrosis factor-α
WB: Western blot
